# Unraveling the Hemodialysis Patient Network: Results from the Social Network and Renal Education (SNARE) Transplant Intervention Clinical Trial

**DOI:** 10.1016/j.xkme.2026.101511

**Published:** 2026-08-26

**Authors:** Avrum Gillespie, Alec Gayner, Heather M. Gardiner, Edward L. Fink, Hannah Calvelli, Briana Lee, Zoe Pfeffer, Sally Quinn, Louise Enderle, Christina Petyo, Peter P. Reese, Crystal A. Gadegbeku, Zoran Obradovic

**Affiliations:** 1 Division of Nephrology, Hypertension, and Kidney Transplantation, Department of Medicine, Lewis Katz School of Medicine, Temple University, Philadelphia, PA; 2 Department of Medicine, Perelman School of Medicine, University of Pennsylvania, Philadelphia, PA; 3 Department of Social & Behavioral Sciences, College of Public Health, Temple University, Philadelphia, PA; 4 Department of Communication and Social Influence, Klein College of Media and Communication, Temple University, Philadelphia, PA; 5 Department of Surgery, Perelman School of Medicine, University of Pennsylvania, Philadelphia, PA; 6 Department of Medicine, Oregon Health & Science University, Portland, OR; 7 Department of Thoracic Medicine, Lewis Katz School of Medicine, Temple University, Philadelphia, PA; 8 Dialysis Clinics Incorporated, Philadelphia, PA; 9 Vanderbilt Center for Transplant Science, Vanderbilt University Medical Center, Vanderbilt University, Nashville, TN; 10 Glickman Urological and Kidney Institute, Cleveland Clinic, Cleveland, OH.; 11 Center for Data Analytics and Biomedical Informatics, Temple University, Philadelphia, PA

**Keywords:** Educational intervention, hemodialysis, living donor kidney transplantation, social networks

## Abstract

**Rationale & Objective:**

Patient social networks in in-center hemodialysis (HD) facilities may disseminate kidney transplant (KT) information and behaviors. Previously, patients in clustered networks (highest proportion of network members connected with each other [clustering]) completed more KT steps than patients who were central hubs with many network connections (centrality). We hypothesize that participants in clustered networks will more effectively disseminate the information and behaviors of the COmmunicating About Choices in Transplant (COACH) intervention than participants central to the network.

**Study Design:**

The Social Network and Renal Education (SNARE: Promoting Transplantation) was a double-blind nonrandomized network intervention trial using parallel assignment in which participants on the Monday, Wednesday, Friday (MWF) shifts were assigned to the centrality arm whereas those on the Tuesday, Thursday, Saturday (TTS) shifts were assigned to the clustering arm.

**Settings & Participants:**

Transplant-eligible patients at a Mid-Atlantic HD facility whose networks were previously mapped were eligible.

**Intervention:**

Four participants from MWF with the highest betweenness centrality and four participants from TTS with the highest clustering coefficient were selected to be information seeds and participate in and disseminate the COACH to their respective arms.

**Outcomes:**

The number of transplant evaluation steps completed, change in transplant knowledge, and living donation (LD) requests were measured 9 months postintervention for all participants.

**Results:**

Twenty-two transplant-eligible patients, including 7 who received the COACH, completed the study. At 9 months, the clustering arm (*n* = 13) completed more KT evaluation steps than the centrality arm (*n* = 9; 0.92 ± 1.71 vs –0.44 ± 1.24, *P* = 0.04). LD requests were not different (3 vs 1, *P* = 0.88).

**Limitations:**

The coronavirus disease (COVID)-19 pandemic significantly reduced our sample size. Fewer participants in the centrality arm were connected. Arm assignment was not random.

**Conclusions:**

In this novel HD patient network intervention, clustered patients may have greater influence on transplant behaviors than central patients.

## Plain-Language Summary

Hemodialysis facilities are unique treatment environments where patients form relationships with each other creating a patient social network. These networks have the potential overcome barriers to living donor kidney transplantation by providing correct knowledge, communication skills, and reinforcing positive transplant behaviors. Based on our previous observations, we developed a clinical trial to test which network member feature best disseminates a living donor transplant communication intervention. We found that network members whose members were also connected with each other (clustered) positively affect transplant behaviors but not transplant knowledge compared with those who have the most access to information (betweenness centrality). The study had multiple setbacks because of the coronavirus disease (COVID)-19 pandemic but illustrates the potential for social network intervention in hemodialysis facilities.

Improving access to living donor kidney transplantation (LDKT) is a priority because most people with kidney failure are treated with in-center hemodialysis (HD), which is associated with high rates of morbidity and mortality.^1^, ^2^, ^3^ Despite the survival benefits of LDKT, it is underused, particularly among patients from historically marginalized backgrounds.^4^, ^5^, ^6^, ^7^ Although many patient-related barriers to LDKT are structural (eg, inadequate pretransplant health care and education, delayed referrals, and prolonged evaluation), 2 major patient-level barriers have been identified: (1) completing the steps needed to be waitlisted for a kidney transplant (KT) and (2) making a request for living donation (LD).^4^, ^5^, ^6^, ^7^, ^8^, ^9^, ^10^, ^11^ Interventions to address these barriers are often implemented at transplant centers, and the question remains whether these interventions could be more effective if patients are exposed via patient peers at the HD clinic.^12^^,^^13^

Although Sullivan et al^14^ showed that formal peer mentoring at the HD clinic increased the completion of patient’s KT steps (eg, [1] suitability, [2] interest, [3] referral call, [4] first visit, [5] workup, [6] candidate, [7] active on the waitlist, [8] transplant), research on the impact of informal peer relationships within the HD clinic on KT behaviors is limited.^15^ In-center HD is a unique therapy occurring in a group setting, facilitating patient social network formation through which information and behaviors may spread.^15^, ^16^, ^17^, ^18^ Previously, these HD patient networks have been associated with attitudes toward arteriovenous fistula placement, completing the KT steps, and LD discussions.^16^, ^17^, ^18^

Identifying network members and structures which best promote health behaviors is an active research area. Public health social network interventions have shown that informal peer networks enhance recruitment for health-related activities.^19^^,^^20^ Paradoxically, they showed that network members with the most relationships and could reach the most people (defined as degree centrality) were not as effective at disseminating health-related interventions as network members identified as friends without having high degree centrality.^19^

It is unknown which HD network characteristic is the most influential. Betweenness centrality has been associated with positive KT information and attitudes.^21^, ^22^, ^23^ Betweenness centrality is a network measure that calculates if information were to spread through the network, who would it have to pass through the most to reach all the network members (Fig 1).^24^^,^^25^ It is possible that having access to and serving as a conduit or gatekeeper for novel information increases positive KT information and attitudes.^24^^,^^25^ Another network measure, namely, a high clustering coefficient, which represents a high proportion of actual relationships from the pool of all possible relationships in the network (Fig 1), has been associated with completing more KT steps than centrality.^16^ This clustering may form influential echo chambers in which patient shared similar information with each other and reinforce the complex KT behaviors.

**Figure 1:**
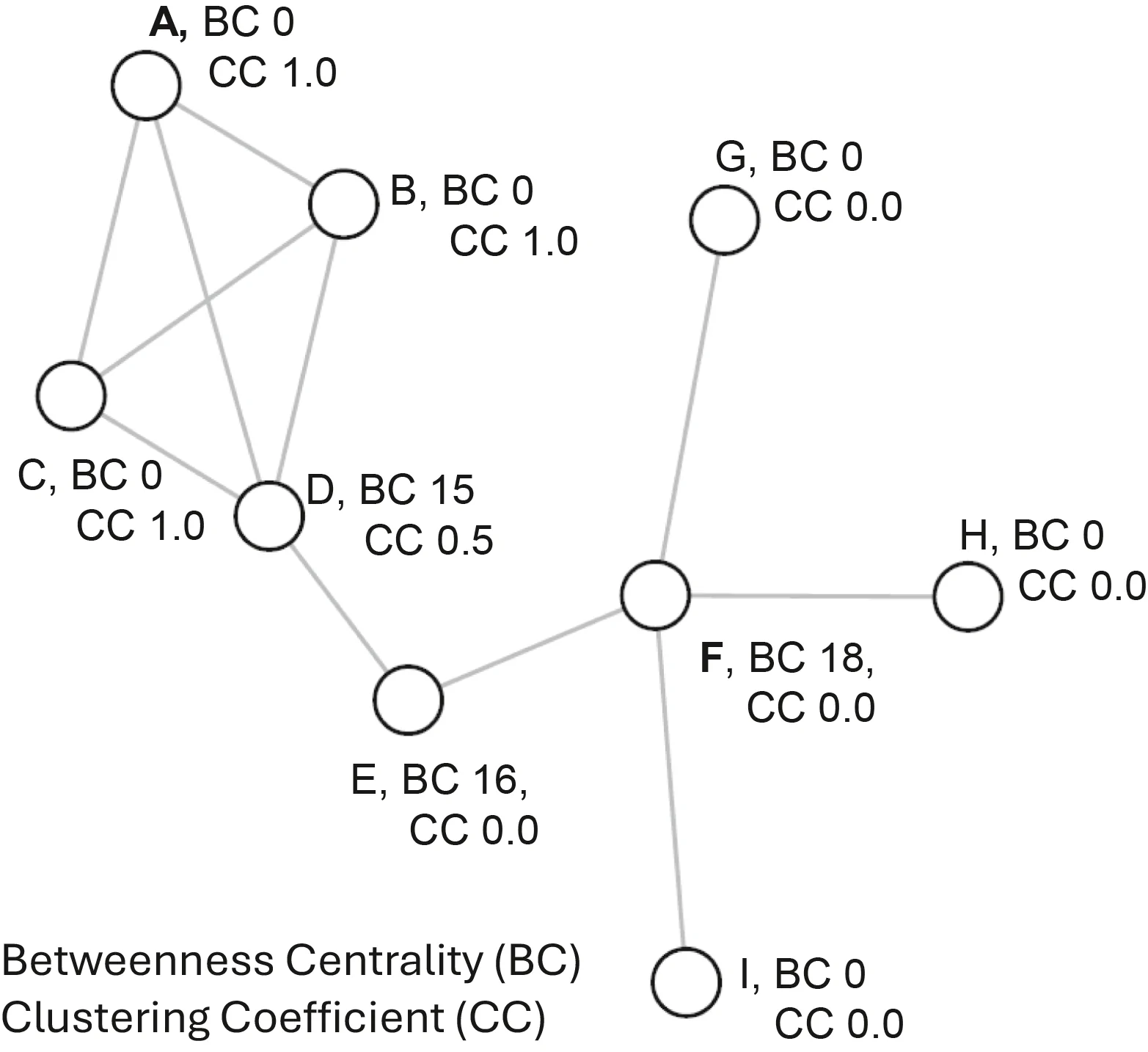
Kite network demonstrating betweenness centrality versus clustering coefficient. Figure 1 shows a model “Kite” network to demonstrate betweenness centrality (equation 1) and clustering coefficient (equation 2). In this network diagram, each node (circle) represents a patient and a line indicates a relationship between them. Nodes are labelled A-I, the first number after the letter is the betweenness centrality, and the second number is the clustering coefficient. Node F has the highest betweenness centrality as in order for nodes G-I to give or get any information, it must pass through node F. Nodes A-C have 0 betweenness centrality because information could pass from A to D directly or indirectly through B or C. G-I have 0 betweenness centrality as they are only connected to F. A-C have clustering coefficients of 1.0 because all (100%) of their network members are connected with each other. D has a clustering coefficient of 0.5 because 50% of the possible connections between the network members exist as there are no connections between E and A-C. We hypothesize that intervening on the most clustered patient A will have a greater informational and behavioral effect on the network compared with intervening on patient F, the one with the highest betweenness centrality. Equation 1. Betweenness Centrality = BC_*i*_ = $\Sigma_{s\neq v\neq t}\frac{\sigma_{st}\left(v\right)}{\sigma_{st}}$. Betweeness centrality of network member v is the sum of the total number of shortest paths from network member s to network member t that pass through v = $\sigma_{st}\left(v\right)$, divided by the total number of shortest paths between network member s and network member t = $\sigma_{st}$. Equation 2. Clustering Coefficient = CC_*i*_ = $\frac{2e_{i}}{k_{i}\left(k_{i}-1\right)}$. Clustering Coefficient of network member i, where $e_{i}$ is the number of observed relationships of network member i and $\frac{k_{i}\left(k_{i}-1\right)}{2}$ is the maximum possible number of relationships that network member i’s network members could have with each other. Abbreviations: BC, betweenness centrality; CC, clustering coefficient.

These previous HD facility studies were observational.^16^^,^^17^^,^^21^, ^22^, ^23^ Thus, to test how HD social network characteristics influence transplant-related attitudes and behaviors, we designed a clinical trial, enrolling transplant-eligible patients in previously mapped HD clinic social networks to examine whether the most clustered or most central patients would disseminate a transplant intervention.^22^^,^^23^^,^^26^ We used the COmmunicating About CHoices in Transplant (COACH) intervention (Item S1), an educational and communication skills intervention previously validated to significantly enhance by improving transplantation-related knowledge and communication skills regarding LDKT and DDKT and LD requests by greater than 20%.^26^ We hypothesized that participants with the highest clustering will more effectively disseminate the information, skills, and behaviors of the COACH intervention than participants with the highest centrality.

## Materials and Methods

### Study design

The Social Network and Renal Education (SNARE: Promoting Transplantation, NCT03536858) was a double-blind nonrandomized trial using a 1:1 parallel assignment. Participants were assigned to their treatment arm based on the day of the week: Monday, Wednesday, Friday (MWF) or Tuesday, Thursday, Saturday (TTS). Four participants (SNARE seeds) on the MWF shifts (2 per AM shift 5 AM to 10 AM and PM shift 10 AM to 3 PM) with the highest centrality measures were selected to receive the COACH intervention, and four SNARE seeds on the TTS (2 per AM and 2 per PM) with the highest clustering measures were selected, resulting in 8 SNARE seeds in total. These network measures were calculated based on their entire networks that included both KT-eligible and -ineligible survey participants.^22^^,^^23^ We anticipated SNARE seeds would disseminate information and behaviors from the COACH intervention within their networks. Thus, all transplant-eligible participants on MWF were assigned to the centrality spread arm (centrality arm) and all transplant-eligible participants on TTS were assigned to the clustering spread arm (clustering arm). Given the coronavirus disease (COVID)-19 pandemic, there were delays and attrition, reducing the trial from 2 centers to 1. The Temple University Institutional Review Board approved the study protocol. Written informed consent was obtained from all participants. The clinical and research activities reported here are consistent with the Principles of the Declaration of Istanbul as outlined in the “Declaration of Istanbul on Organ Trafficking and Transplant Tourism” as well as adherence to the Declaration of Helsinki.^27^^,^^28^ The Social Networks in Health Research (SoNHR) guidelines were used to report this trial.^29^

### Participants, eligibility, and setting

Patients were eligible to participate if they had kidney failure, were ≥18 years old, and could read and understand English (>95% of those in the facility), were present in the HD facility during initial survey periods (July 2018 to June 2020), and transplant eligible.^23^ A study transplant nephrologist determined transplant eligibility via chart review. If transplant eligible but not referred, the transplant nephrologist consulted with the patient’s attending nephrologist to determine whether the participant was an appropriate candidate and reasons the participant had not been referred. We deferred referral to the attending nephrologist. Exclusion criteria were nursing home residence, >79 years of age, active or recent malignancy, inoperable coronary artery disease, or comorbid illness making survival >3 years after transplant unlikely.

Participants completed a baseline HD network survey to determine network centrality and clustering.^22^^,^^23^ This previously described questionnaire, administered 2 years before the intervention, focused on identifying social network members, the strength of relationships, and information shared.^22^^,^^23^^,^^30^ These data were used to calculate the network measures of betweenness centrality (range 0-100) and clustering coefficient (range 0-1). Betweenness centrality was defined as the number of paths connecting any given network member to other members that must pass through the member of interest (Fig 1).^24^ Clustering coefficient was defined as the proportion of actual relationships participants report to the number of possible relationships within the person’s direct network (Fig 1).^13^ This questionnaire also measured self-reported demographic and clinical variables including HD vintage, self-reported health, and comorbid conditions. Eligible participants then completed a preintervention survey 1 year after enrollment. The preintervention and follow-up (3 and 9 months) surveys were used for this analysis. All surveys were interviewer-administered. Participants had the option of completing the survey in the HD facility or via phone outside of the facility. All preintervention surveys were performed via phone because of the COVID-19 pandemic (Item S2, Protocol changes). The pre-and postintervention surveys assessed transplant knowledge with 12 true/false questions which had been developed specifically for the COACH intervention.^26^ In all surveys, participants were queried about whether they had requested an LD and, if so, how many people had they requested an LD from.

### Intervention assignment

After the preintervention surveys were complete, 4 participants (SNARE seeds) with highest betweenness centrality measures on the MWF shift and 4 participants (SNARE seeds) with the highest clustering coefficient on the TTS shifts received the COACH intervention (Table S1).

The COACH intervention is an educational and behavioral communication intervention designed specifically for kidney failure patients pursuing kidney transplantation with the goal of enhancing participant’s knowledge and communication skills regarding living and deceased donor kidney transplantation. The development, content and validation of the COACH intervention has been described elsewhere.^26^ The multimedia intervention was administered via the phone by 1 of 2 research nurses with hemodialysis, kidney transplant, and health care education experience. The intervention contains 4 modules, each administered for 1 hour during their HD treatment. Patients received the accompanying handbook and video, which illustrated an effective discussion of and request for LD. SNARE seeds were encouraged to share the COACH materials (booklet and video) with family and other clinic patients.

Participants received $10 each time after completing the baseline, preintervention, and 3-month postintervention surveys, and $30 for completing the 9-month postintervention survey ($60 in total). Additionally, SNARE seeds received $30 for completing the intervention, a COACH workbook and DVD, and headphones to listen and speak while receiving the COACH teaching.

## Outcomes

The primary outcomes were (1) change in patients’ KT knowledge and (2) change in KT steps completed; both were assessed at 3 and 9 months postintervention. Change in patient kidney transplant knowledge was measured using the number of correct answers to 12 true-false questions.^26^ KT steps as proposed by Sullivan et al^14^ and used in our previous study were defined on a scale from 1 to 8: (1) suitability for referral to a transplant center, (2) interested in transplantation, (3) referral call to a transplant center, (4) visited a transplant center, (5) completion of transplant center workup/evaluation, (6) deemed successful transplant candidate, (7) on a waiting list or evaluating potential living donor(s), and (8) received a transplant.^16^

Secondary outcomes included completion of KT workup at 3- and 9-months post intervention and self-reported LD requests. Completion of KT steps data were obtained from the HD clinic and transplant center (91% of participants were evaluated at the same center) rather than self-report.^11^ Participants who became transplant ineligible over the course of the study, whether because of death (*n* = 7), severe decline in health (*n* = 2), refusal by the center (*n* = 2) were censored such that their final step completed before ineligibility was used in the analyses. Participants deactivated for not completing their annual evaluation (not medical reasons) were penalized 1 step.

### Sample size, blinding, and statistical analyses

#### Sample size

The power analysis was revised preintervention to assess viability because of low enrollment caused by the COVID-19 pandemic (see Supplementary Material). Based on previous observations, the most clustered members completed 2 more KT steps in the transplant than the most central (7 ± 1.5 steps vs 5 ± 1.7 steps, *P* = 0.03).^16^ Thus, we determined a total sample of 22 (11 in each treatment arm) would yield 80% power at significance level α = 0.05 to detect a difference of 2.2 ± 1.7 transplant steps (effect size of 1.25).

#### Blinding

SNARE seeds were unaware that they were selected based on network measures. Other participants, HD staff, COACH educators (research nurses), survey administrators, and the primary data analyst were similarly blinded to centrality or clustering arm assignment.

#### Statistical methods

Students *t* tests were used for baseline quantitative variables such as transplant steps completed, change in knowledge scores, and number of living donor requests. Fisher exact and χ^2^ tests were used for categorical variables. A repeated measures analysis of variance was also performed to assess change in transplant steps completed as a function of time (Item S2, Protocol changes). GPOWER was used for the power analysis.^31^ UCINET, statnet, and igraph were used for network analyses and visualizations.^32^, ^33^, ^34^

#### Missing data

Given the COVID-19 pandemic and challenges with administering the survey by phone, not all participants participated in the preintervention knowledge questionnaire and were excluded from the change in knowledge and self-efficacy analyses.

#### Sensitivity analyses

Sensitivity analyses were conducted comparing steps completed by SNARE seeds by treatment arm. We also conducted sensitivity analyses excluding SNARE seeds. We examined for sex differences in LD behavior and completion of transplant steps. We also compared the number of correct knowledge questions by level of education.

## Results

### Participant flow

Figure 2 and Item S4 describe the full participant flow. In brief, of the 33 eligible participants (Table S1), 22 completed 9 months of follow-up and were included in the final analysis with nine in the Centrality Arm and thirteen in the Clustering Arm (Fig 2). Reasons for not completing follow-up included 7 unrelated deaths (including one of the SNARE seeds), 2 participants had severe decline in health, and 2 declining to further study participation. Preintervention surveys were completed in August 2020, and the COACH intervention was administered to 8 participants and completed by September 2021. Follow-up surveys were administered in January-February (4-5 months postintervention) and May-June of 2022 (9 months postintervention) and thus will be termed 3 and 9 months postintervention (see Item S2, Protocol changes). Data collection ended July 1, 2022.

**Figure 2:**
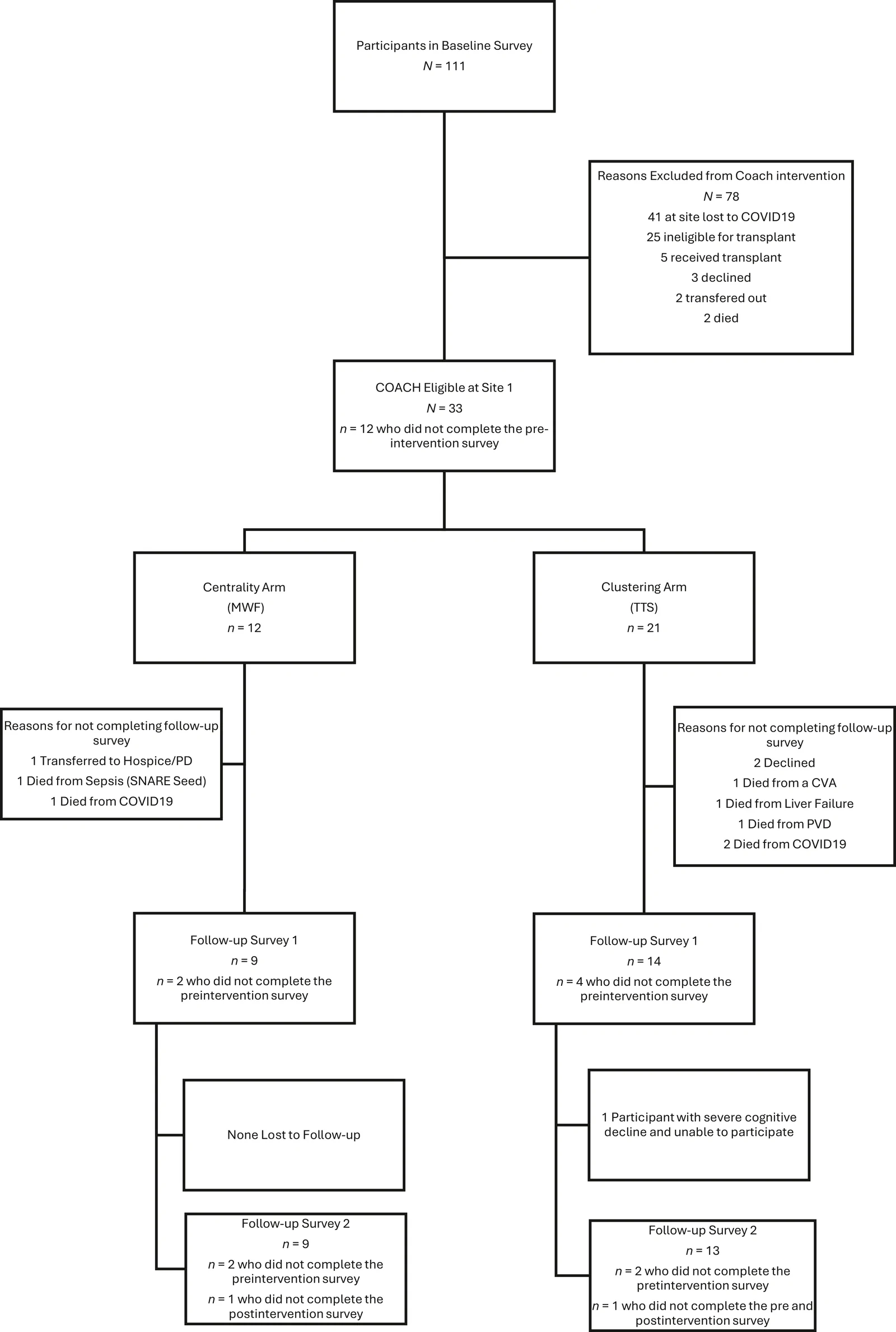
Flow diagram of participants. Figure 2 shows the flow from the initial participants who completed the baseline survey to the initial eligible participants and the number in each of the treatment arms. It also shows which patients did not complete the preintervention survey and the reasons patients were excluded from the final analysis.

### Demographics

#### Demographic and clinical characteristics of SNARE participants

The treatment arms did not differ significantly in demographic variables including age, sex, or race with all participants’ self-reported race as African American or Mixed Race African American (Table 1). Participants did not differ significantly in self-reported clinical characteristics (Table 1). Both intervention groups completed a mean of 4 transplant steps prior to intervention, with none who were active on the transplant waitlist in the centrality arm and 15% in the clustering arm. Most participants (86%) had not made an LD request.

**Table 1:** Baseline Characteristics of the Treatment Arms^a^

Variables	Centrality Arm (N = 9)	Clustering Arm (N = 13)	Total (N = 22)
Demographic Variables			
Age (y)			
Mean (SD)	55.7 (11.7)	54.4 (7.72)	54.9 (9.29)
Female	6 (66.7%)	9 (69.2%)	15 (68.2%)
Race			
African American	9 (100%)	12 (92.3%)	21 (95.5%)
More than one	0 (0%)	1 (7.7%)	1 (4.5%)
Hispanic	0 (0%)	1 (7.7%)	1 (4.5%)
College education (Yes)	4 (44.4%)	8 (61.5%)	12 (54.5%)
Full-time employment	0 (0%)	2 (15.4%)	2 (9.1%)
Income over $20,000	2 (22.2%)	9 (69.2%)	11 (50.0%)
Married or cohabiting	2 (22.2%)	1 (7.7%)	3 (13.6%)
Attend religious service more than once a month	4 (44.4%)	8 (61.5%)	12 (54.5%)
Clinical Variables			
Hemodialysis vintage			
<1 y	3 (33.3%)	1 (7.7%)	4 (18.2%)
1-5 y	4 (44.4%)	5 (38.5%)	9 (40.9%)
>5 y	2 (22.2%)	7 (53.8%)	9 (40.9%)
Health Status			
Excellent/very good/good	4 (44.4%)	7 (53.8%)	11 (50.0%)
Fair/poor	5 (55.6%)	6 (46.2%)	11 (50.0%)
Cancer	2 (22.2%)	2 (15.4%)	4 (18.2%)
Heart disease	1 (11.1%)	3 (23.1%)	4 (18.2%)
Poor circulation	2 (22.2%)	0 (0%)	2 (9.1%)
Lung disease	0 (0%)	2 (15.4%)	2 (9.1%)
No medical conditions	5 (55.6%)	8 (61.5%)	13 (59.1%)
Transplant-Related Variables			
Baseline transplant step			
Mean (SD)	4.6 (1.7)	4.1 (2.1)	4.3 (1.9)
Active on the waitlist	0 (0%)	2 (15.4%)	2 (9.1%)
Requested a LD prior	0 (0%)	3 (23.1%)	3 (13.6%)
Received a prior LD offer	5 (55.6%)	9 (69.2%)	14 (63.6%)
Trust Doctors			
A lot/moderate	7 (77.8%)	12 (92.3%)	19 (86.4%)
A little/not at all/unsure	2 (22.2%)	1 (7.7%)	3 (13.6%)
Importance of Kidney Transplant			
Very important	7 (77.8%)	12 (92.3%)	19 (86.4%)
Somewhat important	2 (22.2%)	1 (7.7%)	3 (13.6%)
Network Measures			
Clustering coefficient mean (SD)	0.28 (0.09)	0.30 (0.18)	0.29 (0.20)
Betweenness centrality median (IQR)	0 (64)	5 (9)	4 (56)

Abbreviations: SD, standard deviation; IQR, interquartile range.

a Table 1 shows the baseline demographic and clinical characteristics of the study population by treatment arm. Age is shown as a continuous variable. The demographic variables were race (participants could select more than one), ethnicity (Hispanic), sex (female, male), annual income (categorical), and education level (categorical). Self-reported health which a five-category variable was recategorized in Excellent/Very Good/Good and Fair/Poor. The medical comorbid conditions of cancer, heart disease, lung disease, poor circulation were self-reported and could reply yes to more than one. If a patient had none of the aforementioned conditions they were labeled as no medical conditions that would be relative contraindications to kidney transplantation. Trust in medical doctors was originally a five-category variable which was recoded to a binary variable. For the question of how important is a kidney transplant, this question had five categories; however, participants only replied as very important or somewhat important. Network Measures were calculated from baseline network data.

#### Demographic and clinical characteristics of SNARE seeds

SNARE seeds did not differ significantly in baseline demographic variables (Table S2). Seeds chosen for their centrality (mean betweenness centrality 90.1 ± 62.0) included 2 females and 2 males. All identified as African American, half were college educated, and half had a reported income over $20,000 per year. Seeds chosen in the clustering arm included 1 self-identified male and 3 self-identified females (mean clustering coefficient 0.44 ± 0.06). All self-identified as African American or mixed race with one seed also identifying as Hispanic, 3 seeds were college educated, and 3 had an income over $20,000 per year (Table S2).

### Primary outcomes

Over 90% (20 out of 22) of participants were evaluated at the same transplant center. Participants in the clustering arm (N = 13) completed an average of 1.4 more transplant steps over the course of the study as compared with those in the centrality arm (0.92 ± 1.71 vs –0.44 ± 1.24, *P* = 0.04) (Tables 2, S3, and S4; Figs 3, 4, and S1). The size of the nodes in Figure 3 shows the change in the number of steps completed with the larger nodes completing more steps post intervention. No significant differences were observed in the change in knowledge scores 9 months postintervention between the clustering and centrality arms (–1.40 ± 2.84 vs –0.83 ± 1.17, *P* = 0.59, Table 3). Table S5 displays knowledge items and their changes from before and after the intervention. Notably, after the intervention, only 63% in the centrality arm and 38% in the clustering arm believed that patients who receive a kidney transplant live longer than people receiving dialysis (Question 12).

**Table 2:** Change in Transplant Steps Postintervention^a^

Change in StepsMean (SD)	Centrality Arm (N = 9)	Clustering Arm (N = 13)	*P* value
Baseline -> 3 months	0.11 (0.33)	0 (1.47)	0.80
3 months -> 9 months	-0.56 (1.01)	0.92 (1.38)	0.009
Baseline -> 9 months	-0.44 (1.24)	0.92 (1.71)	0.04

a Table 2 shows the primary outcome of difference steps completed between the baseline preintervention survey and 3 months postintervention, 3 months postintervention to 9 months postintervention (9 months), and from the baseline preintervention to 9 months post intervention. Participants could “lose” steps if they were inactivated from the waitlist or were found ineligible if they did not complete their transplant evaluation.

**Figure 3:**
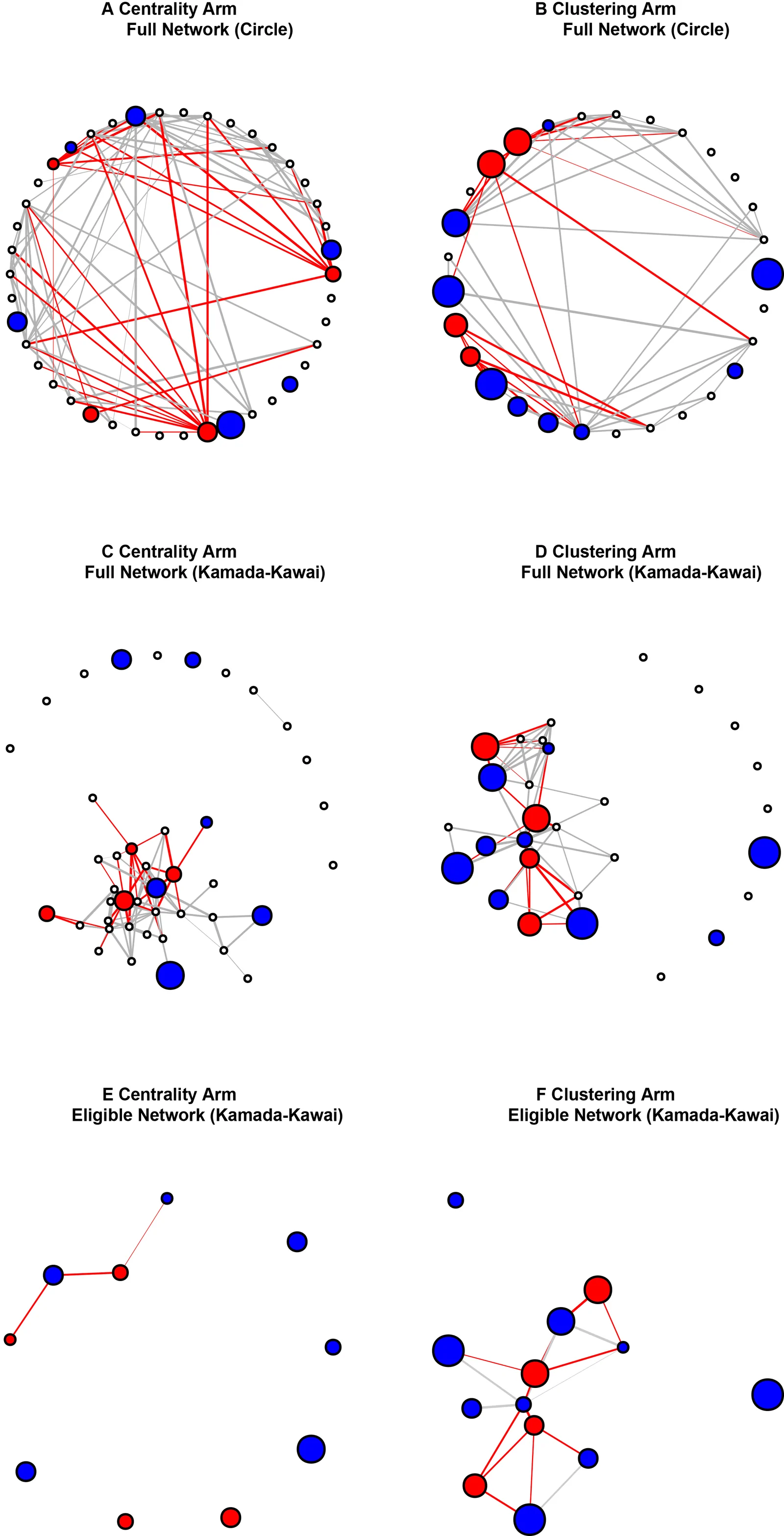
Hemodialysis patient social networks. Figure 3 shows the hemodialysis patient social network by treatment arm. The nodes represent the patients. The red nodes represent the SNARE seeds, and the blue nodes represent the eligible participants. The small dark black ones represent ineligible participants. The size of the nodes represents the change in steps before and after the intervention. A red line between nodes indicates a relationship with a SNARE seed. A gray line represents a relationship between those who are not SNARE seeds. Panels A and B are in the circle layout and include those who are ineligible for transplant. This was selected to best visualize the number of relationships that the SNARE seeds have. Panels C and D are in the Kamada–Kawai layout (ref) that places those who are central in the center of the figure and those without relationships (isolates) out to the periphery. Additionally, if network members share multiple relationships (clustering) they are placed closer to each other. These panels include those who are ineligible and reflect the network measures by which the SNARE seeds were selected. Panels E and F show the networks when the ineligible members are dropped. Note that only 4 members are connected directly in the centrality arm. Thus, for information to travel, it must go through ineligible members. Lastly, these figures show the one SNARE seed who passed away before follow-up and was not included in the final analysis.

**Figure 4:**
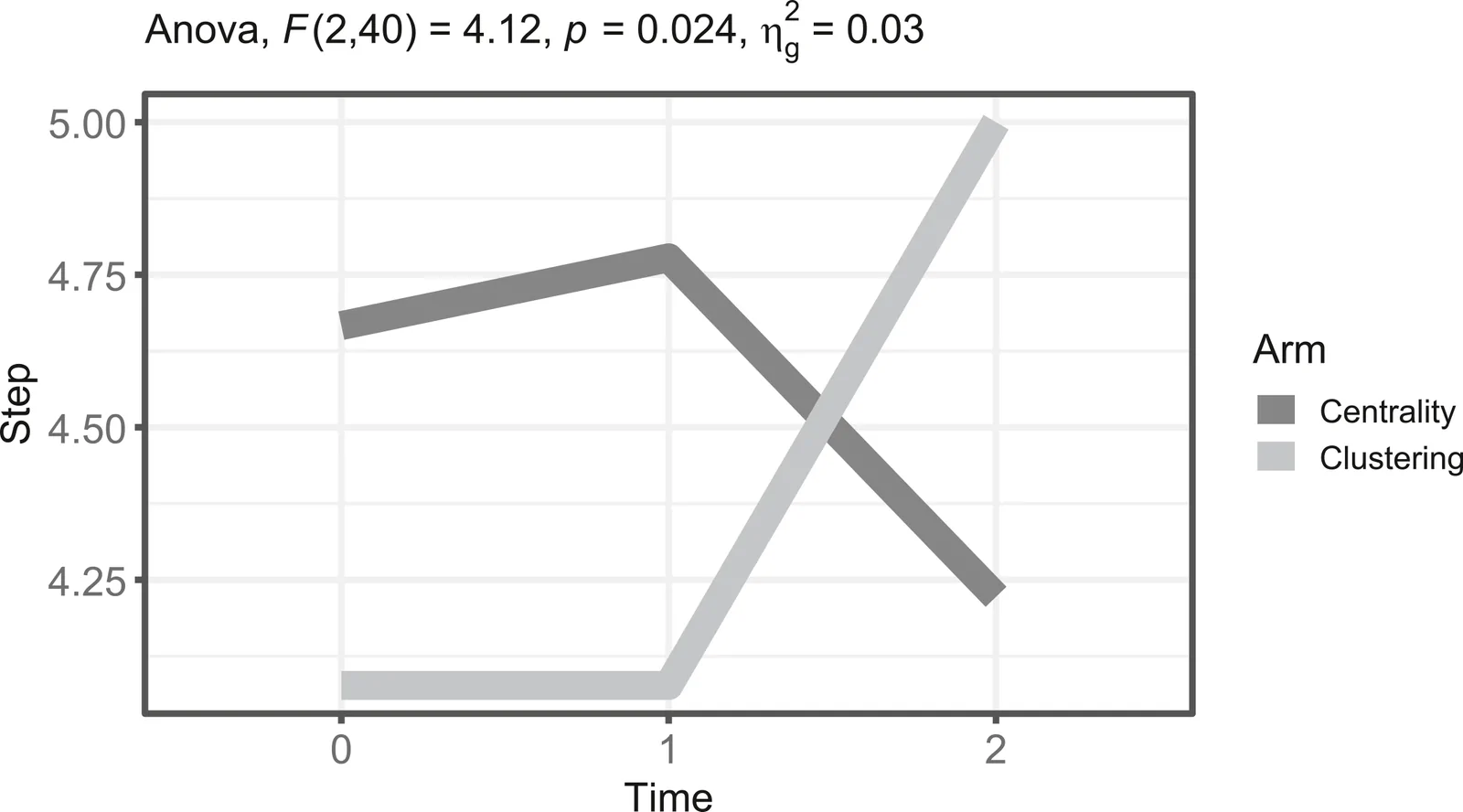
Mean step in transplant process over time by treatment arm. Figure 4 shows the mean step in the transplant process over time between the treatment arms. The centrality arm is in dark gray whereas the clustering arm is in light gray. Time 0 is preintervention, time 1 is the 3 month follow-up, and time 2 is the final collection 9 months postintervention. The results of the repeated measures ANOVA is shown above. Participants could “lose” steps if they were inactivated from the waitlist or were found ineligible if they did not complete their transplant evaluation. Abbreviation: ANOVA, analysis of variance.

**Table 3:** Transplant Knowledge at Baseline, Post intervention, and Change Over 9 Months^a^

Number of Correct True/False Transplant Knowledge Questions	Centrality Arm (*n* = 9)	Clustering Arm (*n* = 13)	Total (*n* = 22)	*P* value
Baseline				
Mean (SD)	6.71 (2.36)	7.40 (1.96)	7.12 (2.09)	0.54
Missing	2 (22.2%)	3 (23.1%)	5 (22.7%)	
Final (9 Months Post)				
Mean (SD)	6.63 (1.06)	5.75 (2.14)	6.10 (1.80)	0.24
Missing	1 (11.1%)	1 (7.7%)	2 (9.1%)	
Difference Post-Pre				
Mean (SD)	–0.833 (1.17)	–1.40 (2.84)	–1.19 (2.32)	0.59
Missing	3 (33.3%)	3 (23.1%)	6 (27.3%)	

Abbreviation: SD, standard deviation.

a Table 3 shows patients transplant knowledge based on the sum of the correct answers to 12 true/false questions at time 1 before intervention (baseline) and the final time 9 months postintervention. We also measured the difference between post intervention and preintervention. Missing data is shown for each time period. Missing Data: In the centrality arm, 2 participants did not complete the preintervention survey, and 1 participant, who completed the preintervention survey, did not complete the postintervention survey. Thus, 3 participants were missing from the final analysis. In the clustering arm, 3 participants did not complete the preintervention survey, and 1 participant who did not complete the preintervention survey also did not complete the postintervention survey. Thus, 3 participants were missing from the final analysis.

### Secondary and exploratory outcomes

Eight participants in the clustering arm (61.5%) and five participants (55.6%) discussed kidney transplant with family and friends after the intervention (*P* = 1.00, Table 4). Three participants in the clustering arm made LD requests (21%) as compared with 1 (13%) in the centrality arm; this was not significantly different (*P* = 0.88, Table 4). There were no differences between arms in the proportion of participants achieving transplant waitlist or receiving a kidney transplant. No participant in the evaluation process (Step 4) or less had completed their KT evaluation by the study end. One SNARE seed (clustering arm) received an LD transplant 3 months post intervention. One participant in the centrality arm received a deceased donor transplant 10 months postintervention.

**Table 4:** Secondary Outcomes of Requesting a Living Donation, Becoming Active on the Kidney and/or Receiving a Kidney Transplant Pre- and Postintervention by Treatment Arm

Variables	Centrality Arm (N = 9)	Clustering Arm (N = 13)	Total (N = 22)	*P* Value^a^^,^^b^
Discussed kidney transplantation with family or friends 9 months postintervention				1.00^a^, 0.11^b^
Yes	5 (55.6%)	8 (61.5%)	13 (59.1%)	
No	4 (44.4%)	5 (38.5%)	9 (40.9%)	
Made a living donor request after the intervention				0.88^a^, 0.11^b^
Yes	1 (11.1%)	3 (23.1%)	4 (18.2%)	
No	8 (88.9%)	10 (76.9%)	18 (81.8%)	
Active on the transplant waitlist or received a kidney transplant 9 months postintervention				0.42^a^, 0.13^b^
Not on the waitlist	8 (88.9%)	8 (61.5%)	16 (72.7%)	
Active on the waitlist or received a transplant^c^	1 (11.1%)	5 (38.5%)	6 (27.3%)	

Note: Table 4 shows the secondary outcomes of making a living donor transplant request and achieving the kidney transplant waitlist and/or receiving a kidney transplant.

a Fisher exact test was used to compare the 2 x 2 table proportions.

b Cochran–Mantel–Haenszel test was used to compare proportion before intervention and 9 months after the intervention.

c One participant in the centrality arm received a deceased donor kidney transplant, and one in the clustering arm who was a SNARE seed received a living donor kidney transplant.

### Subgroup and sensitivity analyses of SNARE seeds

SNARE seeds completed an average of over 2 additional steps toward transplant among the most clustered seeds when compared to the most central seeds (1.25 ± 0.96 vs –1.0 ± 1, *P* = 0.04). SNARE seeds’ knowledge change scores did not differ significantly between the clustering or centrality arms (–0.25 ± 3.77 vs –1.5 ± 0.71, *P* = 0.59; Table S6). Table S7 shows changes in SNARE seeds communication postintervention. When excluding the SNARE seeds, the clustering arm completed more steps than the centrality arm, but this was not significantly different (0.78 ± 1.99 vs –1.7 ± 1.33, *P* = 0.29). There were no significant sex differences in KT steps.

### Harms

There were no harms directly related to this study; however, 7 patients (21%) died, 3 from COVID-19, before completing the first follow-up survey and were excluded from the final analysis. Two participants were excluded from further participation because of declining health unrelated to the intervention.

## Discussion

Our data suggests that intervening on the most clustered individuals in the HD social network, rather than the most central, may have a greater behavioral response to the COACH intervention among all network members in completing transplant steps but not living donor requests. This occurred despite no improvement in knowledge scores. These results must be interpreted cautiously given the COVID-19 pandemic’s effects on sample size and data collection; nonetheless, they show the promise of HD social network interventions.

A prior formal peer mentor HD patient intervention showed an average increase in 1.9 additional KT steps compared with controls.^14^ In our study, SNARE seeds functioned as informal peer mentors and clustering arm participants completed 1.4 more steps than centrality arm participants. This supports our prespecified hypothesis that clustered members influence behaviors more than central members, consistent with our prior 3-year observational study of 42 HD patients.^16^ In that study, HD patients with network members who were fully interconnected (clustering coefficient of 1.0) completed 2.2 more steps than patients whose members were not (clustering coefficient of 0). Participants may have completed more steps in that study because many had recently begun dialysis, whereas in the current study, most participants had a dialysis vintage of over 5 years. Furthermore, 1 year of follow-up may be insufficient given the many structural barriers to completing the transplant evaluation process (eg, being scheduled for and getting to necessary appointments and procedures in a timely fashion) and that no participants early in their transplant evaluation (ie, Step 2) completed their transplant workup.^14^

Clustered networks may influence behaviors through “echo chamber” effects, reinforcing norms through continuous interaction amongst multiple members leading to behavioral change.^35^, ^36^, ^37^ Our network intervention improved transplant step completion but not knowledge dissemination, suggesting behavior change may occur via peer imitation rather than knowledge gain. Network echo chambers may affect behaviors that can be consistently reinforced by peers as compared with knowledge that may wane over time and is perhaps less likely reinforced by network peers. Conversely, central patients may be less motivated to pursue transplant because concerns of position and status.^21^ Future research is needed to determine whether engineered relationships improve outcomes.

The results of this trial must be interpreted with extreme caution. Given the COVID-19 pandemic and attrition, our enrollment was limited to one HD facility resulting in a small sample size. This limits the generalizability of these findings to other HD facilities especially if more than English is spoken.^17^ Although we found no changes in knowledge scores, the COACH previously showed a 2-question improvement.^26^ Participants’ answers were possibly subject to interviewer administration bias, limitations to participants’ attention, and survey fatigue with several participants skipping the knowledge and communication self-efficacy questions. Differences in completion of transplant steps between the arms could have been from other factors such as differences in attrition rates (8 in the clustering arm and 3 in the centrality arm), loss of a SNARE seed in the centrality arm potentially limiting information spread, and differences in network structure in which fewer transplant eligible participants were connected in the centrality arm compared with the clustering arm. This difference in network structure could have affected dissemination as well as reinforcement of transplant behaviors because in order for information to travel to another participant in must past through an ineligible participant who may or may not have had the capacity to transfer the information. Further, although there were no demographic differences between arms, patients were not randomly assigned to their dialysis shift, and there may be unmeasured differences for the reasons patients are assigned to their dialysis shift. For example, it may be easier to maintain employment on the TTS shift. Additionally, although we saw strong participation amongst those in the facility, not all opted to participate. Thus, we were not able to map every patient in the HD unit. Lastly, perhaps a better methodology should be developed to measure transplant steps because these steps are not equidistant. Nonetheless this was the prespecified method, and this method is extremely useful in identifying systematic KT barriers.^14^

Despite these limitations, this trial demonstrates the potential of network interventions in HD facilities. Future studies with larger, more diverse samples may help understand network effects on outcomes such as LD requests and transplantation. Interventions such as seating eligible patients together or targeting entire clusters may enhance behavior change.^17^ Lastly, including family network members may further improve outcomes, as they may more effectively facilitate living donor recruitment and navigate family dynamics.^38^, ^39^, ^40^

In conclusion, clustered networks may promote the transplant step completion in the HD facility by potentially propagating health-related behaviors and that targeting members in clustered networks within the facility may lead to more efficient dissemination of health-related interventions. Further research across multiple HD facilities is needed to examine the influence of demographic and clinical variables on these findings.

## Supplementary Materials

Supplementary File (PDF) Items S1-S4; Tables S1-S7; Figure S1
