# Supplementary material for: Unraveling the Hemodialysis Patient Network: Results from the Social Network and Renal Education (SNARE) Transplant Intervention Clinical Trial
Source: Kidney Med. 2026 Aug 26;8(11):101511. doi: 10.1016/j.xkme.2026.101511 (PMC13671371; doi:10.1016/j.xkme.2026.101511)
Supplement: Supplementary file 1 [file mmc1.pdf]

SUPPLEMENTAL MATERIALS

Table of Contents

Item S1. COACH Description……………………………………………………….…………………….2

Item S1. Protocol Changes……………………….……………………………….…………………....2

Item S3. Revised Sample Size and Power Calculation……..……………………………………….2

Item S4. Full Participant Flow…………………………………………………………………………..4

Supplemental Table 1. Baseline Characteristics of those who did and did not complete the study………………………………………………………………………………………………5

Supplemental Table 2. Baseline Characteristics of the SNARE seeds by Treatment Arm……….7

Supplemental Table 3. ANOVA Table change in the number of steps …………………………….9

Supplemental Table 4. Frequency and Percentage of Participants at each Transplant Step at each Time Period……………………………………………………………………………………..10

Supplemental Figure 1. Participants Step in Transplant Process Over Time by Treatment Arm……………………………………………………………………………………………………..11

Supplemental Table 5. Twelve True/False Kidney Transplant Knowledge Questions Before and After Intervention……………………………………………………………………......................,12

Supplemental Table 6. Change in Knowledge of the SNARE seeds by the Treatment Arms…..13

Supplemental Table 7. Transplant Communication Pre and Post COACH intervention…………14

Item S1. **CO**mmunicating **A**bout **CH**oices in Transplant (COACH) intervention.

The COACH intervention has previously been demonstrated to enhance participant transplantation related knowledge and improve conversation self efficacy in regards to kidney transplantation.(25) The multimedia intervention includes 4 one-hour modules related to kidney transplantation (1) Kidney transplant options, (2) Discussing your transplant options, (3) Requesting living donation, and (4) Maintaining positive relationships.. The intervention was found to enhance KT related knowledge and communication self-efficacy regarding transplant discussions.

Item S2. Protocol changes

The preintervention survey was originally supposed to occur one year after the baseline (instead of two years); however, this was delayed initially by slow recruitment and further delayed by the COVID-19 pandemic restrictions. Furthermore, these surveys were to be in-person but had to be switched to phone-based interviews because of COVID-19 restrictions in the HD clinic. This also significantly delayed the time to complete the pre-intervention surveys. Pre-intervention survey was completed in August of 2020, the COACH intervention was administered to 8 participants June-September 2021. Follow up surveys administered in January-February and May-June of 2022. It was prespecified that participants would be followed 3 months and 12 months post-intervention; however, this timeline was affected by logistics of the COVID-19 pandemic. Data collection ended July 1, 2022.

Item S3. Revised Sample Size and Power Calculation

The original power calculation was based on the data from our previous HD clinic social network study where we found that the most clustered patients completed 2 more steps in the transplant work-up than the most central (7 ±1.5 steps vs. 5 ± 1.7 steps, p=0.03). The observed effect size was 1.25 or a difference of 2 steps. Thus, we would anticipate that the Clustered

intervention arm would complete 2 more steps than the Centrality arm. We estimated at least 10 patients per shift would be exposed to the intervention either directly through participation in the intervention or indirectly through information/behavior spread through the social network. Both clinics have 6 shifts, and we would have 60 patients assigned to each intervention condition. Based on a two tailed t-test, a total sample of 30 (15 in each treatment arm) would yield 80% power at significance level α = 0.05 to detect a difference of 2 steps. With 120 patients (60 patients in each treatment arm) we will be powered to detect a difference of 1 step. The COACH intervention produced a change in knowledge with an effect size of 1.2, thus if we expose 90 patients (45 in each arm) to the intervention, we will have the power to detect at least half that effect .Before the intervention, we convened the Data Monitoring Safety Board who recommend an updated power calculation. We revised the power calculation for the effect size to be 1.29 (Mean difference 2.2 ±1.7 steps), thus we would need 22 participants to achieve 80% power at a significance level α = 0.05.

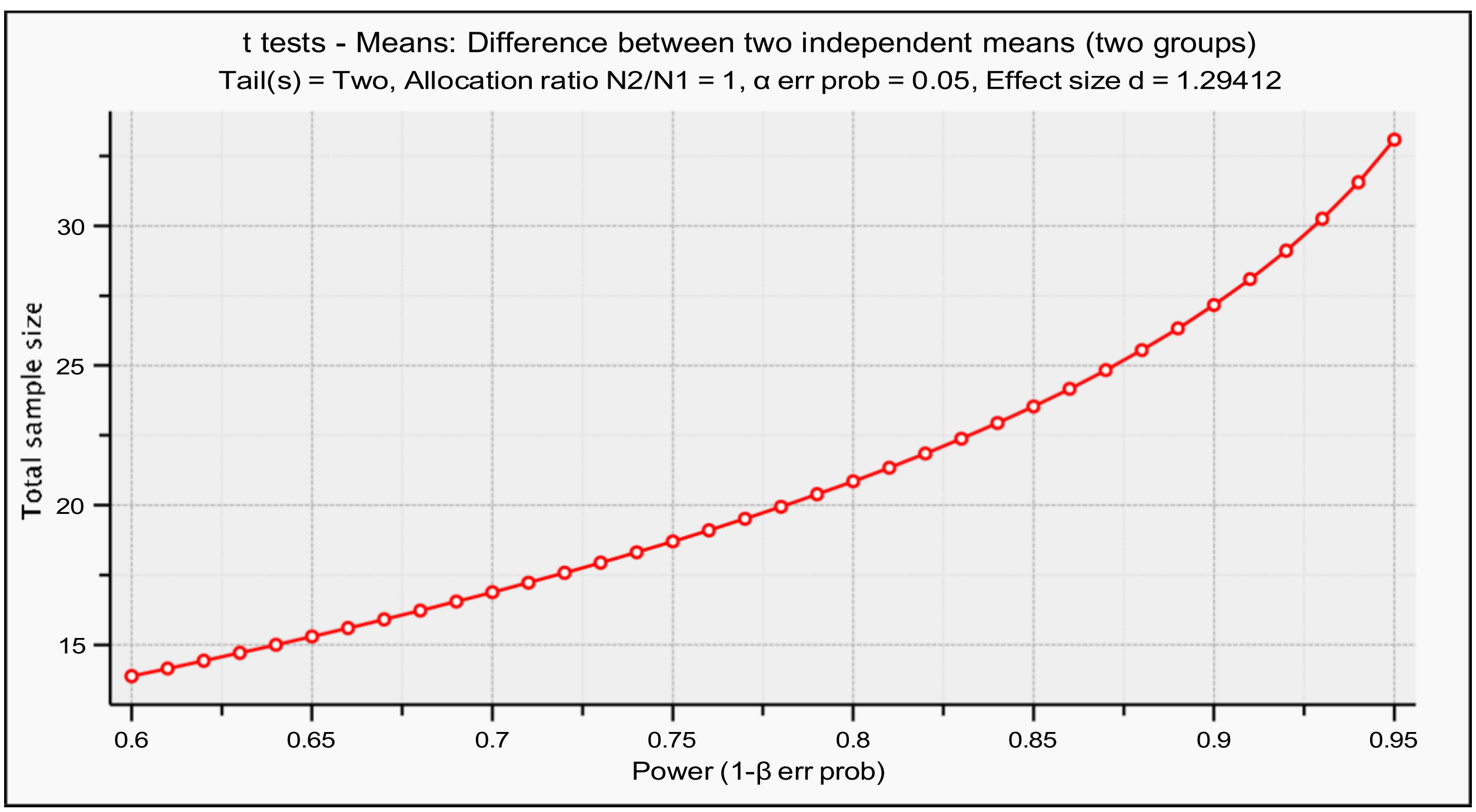

Item S4. Full Participant Flow

Seventy patients at the one dialysis facility completed the baseline social network survey approximately two years prior to the intervention. In the intervening time, twenty- five were found to be ineligible for the kidney transplant waitlist, five who received a kidney transplant, three who declined to participate, and two who died. Of the thirty-three eligible participants, of whom twenty-two participants completed nine months of follow-up and were included in the final analysis with nine participants in the Centrality Arm and thirteen in the Clustering Arm (Figure 2). Reasons for not completing follow-up included seven unrelated deaths (including one of the SNARE seeds), two participants with severe decline in health (precluding them from advancing their transplant workup), and two declining to further participate in the study. Baseline network surveys were administered in October of 2018. Pre-intervention surveys were completed in August of 2021 at which point the COACH intervention was administered to 8 participants and completed by September 2021. The prespecified follow-up surveys were to be 3 months and 12 months post-intervention; however, due to the COVID-19 pandemic, follow-up surveys were administered in January-February (4-5 months post-intervention) and May-June of 2022 (9 months post-intervention) and thus will be termed 3 and 9 months post intervention. Data collection ended July 1, 2022.

Supplemental Table *1*. Baseline Characteristics of Those Who Did and Did Not Complete the Study

	Did Not Complete First Follow-Up (N=11)	Completed Study (N=22)	Total (N=33)
**Treatment Arm**			
Centrality	3 (27.3%)	9 (40.9%)	12 (36.4%)
Clustering	8 (72.7%)	13 (59.1%)	21 (63.6%)
**Age in Years,** Mean (SD)	56.8 (11.4)	54.9 (9.29)	55.5 (9.90)
**Female Sex**	2 (18.2%)	15 (68.2%)	17 (51.5%)
**Race**			
African American	10 (90.9%)	21 (95.5%)	31 (93.9%)
More Than One	1 (9.1%)	1 (4.5%)	2 (6.1%)
**Hispanic**	1 (9.1%)	1 (4.5%)	2 (6.1%)
**Education**			
No	8 (72.7%)	10 (45.5%)	18 (54.5%)
Yes	3 (27.3%)	12 (54.5%)	15 (45.5%)
**Employment**			
No	11 (100%)	20 (90.9%)	31 (93.9%)
Yes	0 (0%)	2 (9.1%)	2 (6.1%)
**Income (dollars)**			
Under 20,000	5 (45.5%)	11 (50.0%)	16 (48.5%)
Over 20,000	6 (54.5%)	11 (50.0%)	17 (51.5%)
**Married**			
No	7 (63.6%)	19 (86.4%)	26 (78.8%)
Yes	4 (36.4%)	3 (13.6%)	7 (21.2%)
**Attend Religous Service**			
More than Once a Month	2 (18.2%)	12 (54.5%)	14 (42.4%)
Less than Once a Month	9 (81.8%)	10 (45.5%)	19 (57.6%)
**Dialysis Vintage**			
< 1 year	2 (18.2%)	4 (18.2%)	6 (18.2%)
1-5 years	7 (63.6%)	9 (40.9%)	16 (48.5%)
>5 years	2 (18.2%)	9 (40.9%)	11 (33.3%)
**Health Status**			
Excellent/Very Good/Good	3 (27.3%)	11 (50.0%)	14 (42.4%)
Fair/Poor	8 (72.7%)	11 (50.0%)	19 (57.6%)
**Cancer**			
Yes	0 (0%)	4 (18.2%)	4 (12.1%)
No	11 (100%)	18 (81.8%)	29 (87.9%)
**Heart Disease**			
Yes	1 (9.1%)	4 (18.2%)	5 (15.2%)
No	10 (90.9%)	18 (81.8%)	28 (84.8%)
**Poor Circulation**			
Yes	1 (9.1%)	2 (9.1%)	3 (9.1%)
No	10 (90.9%)	20 (90.9%)	30 (90.9%)
**Lung Disease**			
Yes	1 (9.1%)	2 (9.1%)	3 (9.1%)
No	10 (90.9%)	20 (90.9%)	30 (90.9%)
**No Medical Conditions**			
Yes	9 (81.8%)	13 (59.1%)	22 (66.7%)
No	2 (18.2%)	9 (40.9%)	11 (33.3%)
**Transplant Step**			
Not on Waitlist	9 (81.8%)	20 (90.9%)	29 (87.9%)
On Waitlist	2 (18.2%)	2 (9.1%)	4 (12.1%)
**Mean Transplant Step**			
Mean (SD)	3.73 (2.28)	4.32 (1.91)	4.12 (2.03)
**Importance of Kidney Transplant**			

	Did Not Complete First Follow-Up (N=11)	Completed Study (N=22)	Total (N=33)
Very Important	5 (45.5%)	19 (86.4%)	24 (72.7%)
Somewhat Important	5 (45.5%)	3 (13.6%)	8 (24.2%)
Not at all Important	1 (9.1%)	0 (0%)	1 (3.0%)

Supplemental Table 1. Shows the baseline demographic and clinical characteristics of those who did complete the 3 month follow-up and those who did.. Age is shown as a continuous variable. The demographic variables were race (participants could select more than one), ethnicity (Hispanic), sex (female, male), annual income (categorical), and education level (categorical). Self-reported health which a five-category variable was recategorized in Excellent/Very Good/Good and Fair/Poor. The medical comorbidities of cancer, heart disease, lung disease, poor circulation were self-reported and could reply yes to more than one. If a patient had none of the aforementioned conditions they were labeled as no medical conditions that would be relative contraindications to kidney transplantation. Trust in medical doctors was originally a five-category variable which was recoded to a binary variable. For the question of how important is a kidney transplant, this question had five categories; however, participants only replied as very important.

Supplemental Table 2. Baseline Characteristics of the SNARE seeds by Treatment Arms.

	Centrality Arm (n = 4)	Clustering Arm (n = 4)	All (N = 8)
**Age (years)**			
Mean (SD)	58.3 (5.68)	48.8 (11.8)	53.5 (9.96)
Median [Min, Max]	60.5 [50.0, 62.0]	48.0 [36.0, 63.0]	56.0 [36.0, 63.0]
**Sex**			
Male	2 (50.0%)	1 (25.0%)	3 (37.5%)
Female	2 (50.0%)	3 (75.0%)	5 (62.5%)
**Race**			
African American	4 (100%)	3 (75.0%)	7 (87.5%)
More Than One	0 (0%)	1 (25.0%)	1 (12.5%)
**Hispanic**			
Yes	0 (0%)	1 (25.0%)	1 (12.5%)
No	4 (100%)	3 (75.0%)	7 (87.5%)
**Education**			
No	2 (50.0%)	1 (25.0%)	3 (37.5%)
Yes	2 (50.0%)	3 (75.0%)	5 (62.5%)
**Employment**			
No	4 (100%)	3 (75.0%)	7 (87.5%)
Yes	0 (0%)	1 (25.0%)	1 (12.5%)
**Income (dollars)**			
Under 20,000	2 (50.0%)	1 (25.0%)	3 (37.5%)
Over 20,000	2 (50.0%)	3 (75.0%)	5 (62.5%)
**Married**			
No	2 (50.0%)	3 (75.0%)	5 (62.5%)
Yes	2 (50.0%)	1 (25.0%)	3 (37.5%)
**Attend Religious Service**			
More than Once a Month	1 (25.0%)	2 (50.0%)	3 (37.5%)
Less than Once a Month	3 (75.0%)	2 (50.0%)	5 (62.5%)
**Dialysis Vintage**			
1-5 years	4 (100%)	2 (50.0%)	6 (75.0%)
>5 years	0 (0%)	2 (50.0%)	2 (25.0%)
**Cancer**			
Yes	0 (0%)	1 (25.0%)	1 (12.5%)
No	4 (100%)	3 (75.0%)	7 (87.5%)
**Heart Disease**			
Yes	1 (25.0%)	0 (0%)	1 (12.5%)
No	3 (75.0%)	4 (100%)	7 (87.5%)
**Poor Circulation**			
Yes	1 (25.0%)	0 (0%)	1 (12.5%)
No	3 (75.0%)	4 (100%)	7 (87.5%)
**Lung Disease**			
Yes	0 (0%)	0 (0%)	0 (0%)
No	4 (100%)	4 (100%)	8 (100%)
**No Medical Conditions**			
Yes	3 (75.0%)	3 (75.0%)	6 (75.0%)
No	1 (25.0%)	1 (25.0%)	2 (25.0%)
**Baseline Transplant Step**			
Mean (SD)	5.75 (0.957)	5.00 (1.63)	5.38 (1.30)
**Transplant Step**			
Not Active or not on the Waitlist	3 (75.0%)	3 (75%)	6 (75.0%)
Active on the Waitlist	1 (25%)	1 (25%)	2 (25%)
**Ever Request a Living Donor**			
Yes	0 (0%)	1 (25.0%)	1 (12.5%)
No	4 (100%)	3 (75.0%)	7 (87.5%)
**Ever Receive Offer**			
Yes	3 (75.0%)	2 (50.0%)	5 (62.5%)
No	1 (25.0%)	2 (50.0%)	3 (37.5%)

	Centrality Arm (n = 4)	Clustering Arm (n = 4)	All (N = 8)
**Importance of Kidney Transplant**			
Very Important	4 (100%)	4 (100%)	8 (100%)
Somewhat Important	0 (0%)	0 (0%)	0 (0%)
**Network Measures**			
Clustering Coefficient Mean (SD)	0.29 (0.15)	0.44 (0.6)	0.36 (0.13)
Betweenness Centrality Median (IQR)	73 (60)	7 (6)	10 (69)

Supplemental Table 2. Shows the baseline demographic and clinical characteristics of the SNARE seeds by treatment arm. Age is shown as a continuous variable. The demographic variables were race (participants could select more than one), ethnicity (Hispanic), sex (female, male), annual income (categorical), and education level (categorical). Self-reported health which a five-category variable was recategorized in Excellent/Very Good/Good and Fair/Poor. The medical comorbidities of cancer, heart disease, lung disease, poor circulation were self-reported and could reply yes to more than one. If a patient had none of the aforementioned conditions they were labeled as no medical conditions that would be relative contraindications to kidney transplantation. Trust in medical doctors was originally a five-category variable which was recoded to a binary variable. For the question of how important is a kidney transplant, this question had five categories; however, participants only replied as very important. Network Measures were calculated from baseline network data. Standard Deviation (SD), Interquartile Range (IQR).

Supplemental Table 3. ANOVA Table change in the number of steps as function of Treatment Arm (Clustering vs, Centrality) and Time.

Effect	DFn	DFd	F	*p*	ges
Arm	1	20	0.046	0.832	0.002
Time	2	40	0.381	0.686	0.003
Arm:Time	2	40	4.115	0.024[a]	0.030

Supplemental Table 3 shows the results of the repeated measures ANOVA of the change in steps as a function of time and assignment to custering arm. DFn is the degrees of freedom for the number of groups. DFd is the degrees of freedom for the number of measures. F is is the F score of the test. ges is the generalized eta square of the variable.

a. The *p* value is 0.029 when corrected for sphericity.

Supplemental Table 4 Frequency and Percentage of Participants at each Transplant Step at each Time Period

Step	Baseline			3 months			9 months (Final)		
	Clustering	Centrality	Total	Clustering	Centrality	Total	Clustering	Centrality	Total
8	0 (0%)	0 (0%)	0 (0%)	1 (7.7%)	0 (0%)	1 (4.5%)	1 (7.7%)	1 (11.1%)	2 (9.1%)
7	2 (15.4%)	0 (0%)	2 (9.1%)	0 (0%)	1 (11.1%)	1 (4.5%)	4 (30.8%)	0 (0%)	4 (18.2%)
6	0 (0%)	3 (33.3%)	3 (13.6%)	1 (7.7%)	2 (22.2%)	3 (13.6%)	0 (0%)	2 (22.2%)	2 (9.1%)
5	6 (46.2%)	4 (44.4%)	10 (45.5%)	6 (46.2%)	4 (44.4%)	10 (45.5%)	1 (7.7%)	0 (0%)	1 (4.5%)
4	0 (0%)	0 (0%)	0 (0%)	0 (0%)	0 (0%)	0 (0%)	4 (30.8%)	2 (22.2%)	6 (27.3%)
3	2 (15.4%)	1 (11.1%)	3 (13.6%)	2 (15.4%)	1 (11.1%)	3 (13.6%)	2 (15.4%)	3 (33.3%)	5 (22.7%)
2	0 (0%)	0 (0%)	0 (0%)	0 (0%)	0 (0%)	0 (0%)	1 (7.7%)	0 (0%)	1 (4.5%)
1	3 (23.1%)	1 (11.1%)	4 (18.2%)	3 (23.1%)	1 (11.1%)	4 (18.2%)	0 (0%)	1 (11.1%)	1 (4.5%)

Supplemental Table 4 shows the frequency and percentage of participants at baseline, 3 month follow-up, and 9 months (the final timepoint). The kidney transplant steps are (1) Suitability for referral to a transplant center, (2) Interested in transplantation, (3) Referral call to a transplant center, (4) Visited a transplant center, (5) Completion of transplant center workup/evaluation, (6) Deemed successful transplant candidate, (7) On a waiting list or evaluating potential living donor(s), and (8) Received a transplant.

Supplemental Figure 1. Participants' Step in Transplant Process Over Time by Treatment Arm

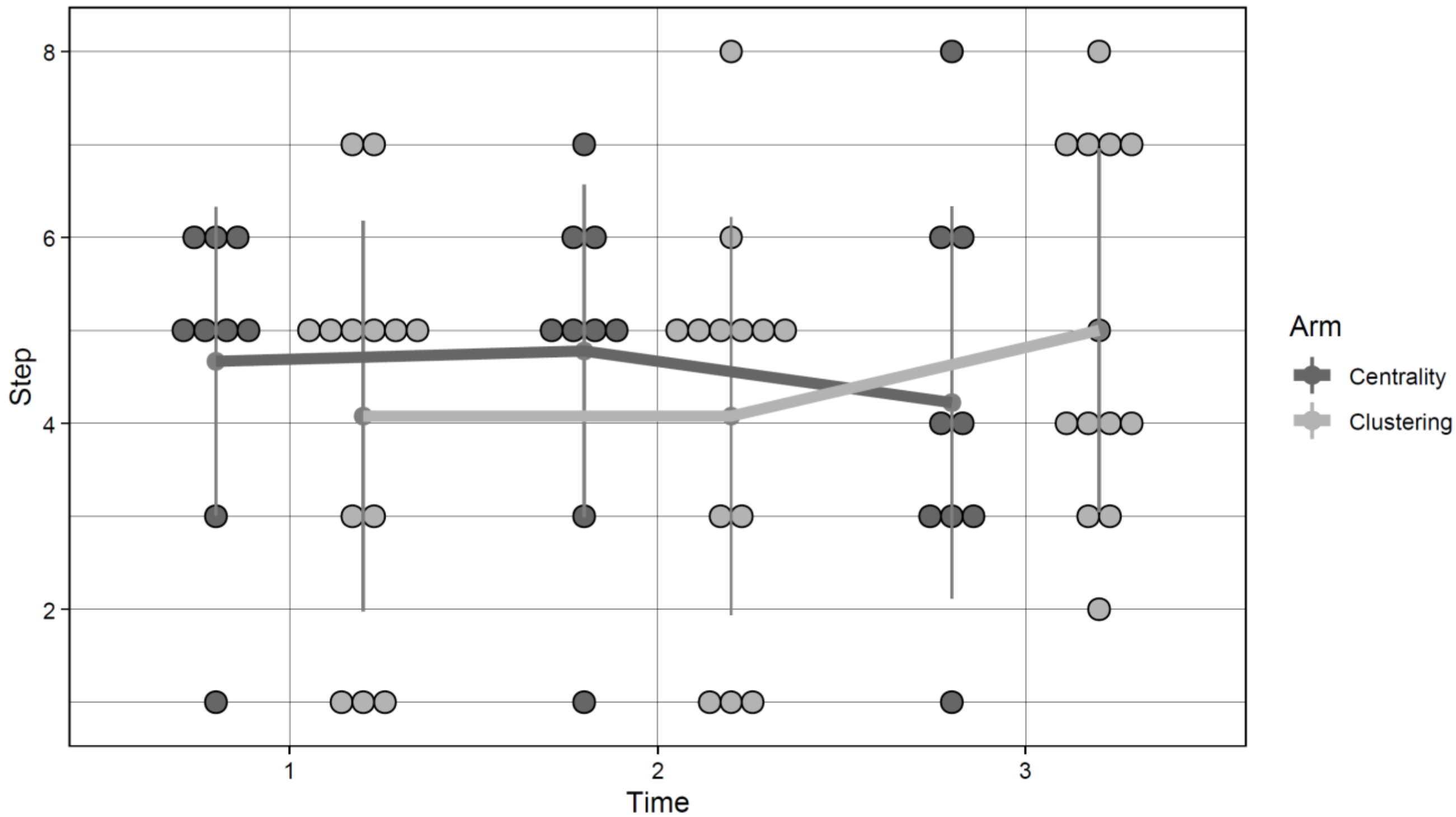

Supplemental Figure 1 shows participants step in transplant process over time by treatment arm

The Centrality Arm is in dark gray whereas the Clustering Arm is in light gray. Each participant is represented by a circle. The thin vertical lines represents the standard deviation and the thick horizontal lines are the mean at each time point. Time 1 is pre-intervention, Time 2 is the 3 month follow-up, and Time 3 is the final collection 9 months post-intervention. Participants could "lose" steps if they were inactivated from the waitlist or were found ineligible if they did not complete their transplant evaluation.

Supplemental Table 5. Twelve True/False Kidney Transplant Knowledge Questions Before and After Intervention.

**True False Knowledge Questions**	Pre-Intervention % Correct		Post-Intervention % Correct		Between Group Differences
	Centrality Group (N= 7)[a]	Clustering Group (N=10)[b]	Centrality Group (N= 8)[a]	Clustering Group (N=12)[b]	*p* value
1. Women may have difficulty with future pregnancies if they donate (False)	57%	60%	55%	46%	0.34
2. Long-term health problems, like high blood pressure or kidney disease, are very rare after living donation (False)	14%	40%	22%	0%	0.29
3. The transplant patient's health insurance will cover the cost of the donors' evaluation, surgery, hospital stay, and follow up care (True)	100%	70%	67%	85%	1.00
4. Living donors must be younger than 50 years old (False)	71%	70%	78%	85%	0.56
5. The risks of live donor surgery are generally small (True)	57%	30%	88%	62%	0.59
6. A living donor's blood and tissue must be a perfect match with the patient (False)	0%	20%	13%	17%	1.00
7. Only healthy adults can be living donors (True)	71%	100%	75%	53%	1.00
8. Most living donors spend only 1 to 3 days in the hospital (True)	71%	90%	75%	69%	0.53
9. Most insurance companies will pay for living donor's indirect costs, like travel, parking, lodging, and lost wages (False)	57%	70%	37%	46%	1.00
10. The success rates of live-donor and decreased-donor kidney transplant are about the same (False)	57%	50%	50%	46%	0.13
11. Patients who receive a kidney transplant from a living donor wait longer for the transplant than patients who receive a kidney from a deceased donor (False)	43%	80%	38%	38%	0.09
12. Patients who receive a kidney transplant live longer than patients on dialysis (True)	71%	60%	63%	38%	1.00

Supplemental Table 5 shows percentage of participants who answered correctly for each of the twelve true/false questions used to test kidney transplant knowledge before and after the intervention. Correct answers to the true/false questions are in parentheses next to the statement. The Cochran-Mantel-Haenszel test was used to calculate the differences in proportions between the two groups. All respondents, not those who completed both pre and post surveys were included in this analysis.

a. In the Centrality Arm, two participants did not fully complete the true/false questions before and one participant after the intervention

In the Clustering Arm, three participants did not complete the true/false questions before and one did not complete it after the intervention.

Supplemental Table 6. Change in Knowledge of the SNARE Seeds by the Treatment Arms.

Number of Correct True/False Transplant Knowledge Questions	Centrality (N=4)	Clustering (N=4)	p value
**Time 1 (Baseline)**			
Mean (SD)	6.75 (2.87)	7.50 (2.38)	0.7
Median [Min, Max]	7.50 [3.00, 9.00]	8.50 [4.00, 9.00]	
**Time 3 (Final Post)**			
Mean (SD)	6.00 (1.41)	7.25 (2.99)	0.53
Median [Min, Max]	6.00 [5.00, 7.00]	8.00 [3.00, 10.0]	
Missing	2 (50.0%)	0 (0%)	
**Difference Post-Pre**			
Mean (SD)	-1.50 (0.707)	-0.250 (3.77)	0.56
Median [Min, Max]	-1.50 [-2.00, -1.00]	0 [-5.00, 4.00]	
Missing	2 (50.0%)	0 (0%)	

Supplemental Table 6. shows the SNARE seeds transplant knowledge based on the sum of the correct answers to 12 true/false questions at Time 1 prior to intervention (baseline) and the Final time 9 months post-intervention. We also measured the difference between post-intervention and pre-intervention. Missing data is shown for each time period note of the SNARE seeds in the Centrality Arm passed away and another was unable to complete the final survey. Standard deviation (SD).

Supplemental Table 7. Transplant Communication Pre and Post COACH intervention

	Centrality (N=4)	Clustering (N=4)	*p* value
**Conversational Self- Efficacy (Pre)**			
Mean (SD)	12.0 (6.06)	13.8 (6.65)	0.711
**Conversational Self-Efficacy (Post)**			
Mean (SD)	18.5 (1.91)	19.5 (5.32)	0.743
**Conversational Self-Efficacy (Change)**			
Mean (SD)	6.50 (7.33)	5.75 (5.97)	0.879

Supplemental Table 7 shows the change in transplant communication before and after the COACH intervention. Improving communication self-efficacy is one of the goals of the COACH intervention. This score is a composite of 9 positive and negative questions. 1) I am satisfied with the outcome of the conversation i had about transplantation; 2) Thinking about holding another discussion about transplantation makes me anxious; 3) I was in control of the conversation the whole time; 4) It was a relief to talk about transplant-related issues with another person; 5) I felt tense and nervous during the discussion about transplantation; 6) My conversation about transplantation was rewarding; 7) I said the things I needed to say to manage the conversation about transplantation effectively; 8) During the discussion about transplantation, I just let the other person do most of the talking; 9) Talking about transplantation was useful and helpful. The questions were answered on a 5 point scale: 1, Strongly disagree | 2, Disagree | 3, Neutral | 4, Agree | 5, Strongly Agree. SNARE seeds significantly improved transplant communication self-efficacy immediately post-intervention (pre; 12.9 ± 6.0 vs. post; 19.0 ± 3.7; $p = 0.03$) but this change did not differ significantly between arms
